# Case Report: Hantavirus Cardiopulmonary Syndrome Diagnostic in the Face of the COVID-19 Pandemic

**DOI:** 10.4269/ajtmh.21-0637

**Published:** 2022-01-19

**Authors:** Elba Regina Sampaio de Lemos, Jorlan Fernandes, Thayssa Alves Coelho, Liana Ogino Lumi, José Antônio Rodrigues Rosa, Letícia Biasus, Zenaida Marion Alves Nunes, Dóris Bercht Brack, Renata Carvalho de Oliveira

**Affiliations:** ^1^ Laboratório de Hantaviroses e Rickettsioses, Instituto Oswaldo Cruz, Fiocruz, Rio de Janeiro, Brazil;; ^2^ Vigilância Epidemiológica, Secretaria Municipal de Saúde, Bento Gonçalves, Rio Grande do Sul, Brazil;; ^3^ Seção de Virologia, Laboratório Central de Saúde Pública, Secretaria Estadual de Saúde, Rio Grande do Sul, Brazil;; ^4^ Divisão de Vigilância Ambiental em Saúde, Secretaria Estadual de Saúde, Rio Grande do Sul, Brazil

## Abstract

Hantavirus cardiopulmonary syndrome (HCPS) is an emerging rodent-borne disease in the Americas. The most common initial symptoms of HCPS are similar to those of COVID-19 and other respiratory infections that evolve rapidly to respiratory failure, resulting from pulmonary edema and shock in about 40% of cases. We describe a fatal case of HCPS in a 24-year-old man who was hospitalized with fever, hemoconcentration, thrombocytopenia, leukocytosis, dry cough and a bilateral diffuse alveolar pulmonary infiltrate during the emergence of the COVID-19 pandemic in Brazil. HCPS needs to be ruled out in patients with clinical manifestations compatible with respiratory infections such as influenza and COVID-19.

## INTRODUCTION

A new and rapidly progressive respiratory syndrome named COVID-19, resulting from infection with severe acute respiratory syndrome coronavirus 2 (SARS-CoV-2), was identified in December 2019 in Wuhan, China, and has become a pandemic.^1^ Currently, almost 226 million cases and more than 4.6 million fatalities resulting from COVID-19 have been reported worldwide. In Brazil, more than 21.1 million cases and 589,000 deaths have been identified so far.^1^^,^^2^ COVID-19 is an acute viral infection with frequent and severe pulmonary involvement that can lead to hospitalization and death. The immune system is also affected markedly and challenged by SARS-CoV-2, and leukopenia, lymphopenia, and an inflammatory cytokine storm are some of the immunological changes.^3^

With many clinical similarities to COVID-19, hantavirus cardiopulmonary syndrome (HCPS) is a severe, acute emerging disease characterized by increased capillary permeability causing vascular leakage, and thrombocytopenia.^4^ The first case of an American orthohantavirus producing human disease cases with predominant pulmonary involvement was reported in the United States in 1993.^5^ The most common initial symptoms are similar to those of COVID-19 and others respiratory infections such as flu, with a febrile prodrome associated with headache, dry cough, muscle pain, and shortness of breath that evolve rapidly to respiratory failure as a result of pulmonary edema and shock^4^^–^^6^ (Table 1). Hemorrhagic complications may occur in cases of HCPS, although severe, disseminated intravascular coagulation is uncommon. Humans are infected primarily via inhaled aerosols of rodent excreta, and reports of person-to-person transmission have been reported in Argentina and Chile associated with the Andes orthohantavirus.^7^^–^^9^

**Table 1:** Epidemiological, clinical, laboratory, and radiographic characteristics of hantavirus cardiopulmonary syndrome and coronavirus disease 2019

Characteristic	Hantavirus cardiopulmonary syndrome	COVID-19
Who is at most risk of infection?	Anyone reporting living or visiting a rural/forestry environment, in contact with rodents or rodent excreta, and opening and cleaning closed-up buildings. Occupational exposure can include agricultural, livestock and forest activities (campers and hikers)	Health-care providers and family members or any close contact of COVID-19 patients are more frequently exposed.
Incubation period	7–60 days	2–14 days
Most common signs and symptoms	Fever, headache, myalgia, headache, vomiting, and diarrhea	Fever, fatigue, cough, shortness of breath, and myalgia
Additional symptoms	Abdominal and/or thoracic pain, dyspnea, cough, and acute respiratory failure	Headache, sore throat, loss off smell, runny nose, bloody sputum, vomiting, and diarrhea
Progression of the disease	Rapid progression to respiratory failure (between days 1 and 6)	Expected worsening between days 7 and 10
Common laboratory findings	Leukocytosis with left shift, neutrophilia, hemoconcentration, and thrombocytopenia	Lymphopenia, thrombocytopenia, and elevated D-dimer levels (significantly more prominent in severe COVID-19 cases)*
Radiographic findings	Unilateral or bilateral pulmonary diffuse interstitial infiltrate and pleural effusions	Consolidation, ground-glass opacities with a peripheral or lower zone distribution and bilateral involvement

Adapted from Oliveira et al.,^15^ de Lacerda Barbosa et al.,^22^ Dai et al. 2020,^25^ and the Centers for Disease Control and Prevention.^29^

* Few studies have investigated between D-dimer levels and Hantavirus cardiopulmonary syndrome common laboratory findings.

Emergence of SARS-CoV-2 in HCPS endemic areas has raised concerns regarding misdiagnosis and inaccurate diagnoses of suspected cases. This report describes the clinical features of a confirmed HCPS during the early stages of the COVID-19 pandemic in Brazil.

## CASE PRESENTATION

In March 2020, a 24-year-old man was admitted to a public health unit in the municipality of Bento Gonçalves, Rio Grande do Sul State, South Brazil, with headache, dry cough, diarrhea, loss of appetite and a 4-day history of fever (37.7–38.8°C), without loss of smell. He reported going on a 2-day trip to Paraná and Santa Catarina 14 days before presentation, and upon returning maintained social isolation according to the health recommendations required during the COVID-19 pandemic. After being clinically evaluated, the patient was discharged with a diagnosis of flu-like syndrome, without laboratory confirmation, and a prescription treatment including the use of anti-inflammatory and analgesic drugs. The following day, approximately 4 hours later, he returned to the health unit complaining of respiratory discomfort associated with dry cough with the presence of blood, tachycardia, intense prostration, nausea, vomiting, and dyspnea.

On admission, the patient’s pulse rate was 125 beats/min, he had a respiratory rate of 32 breaths/min, and his blood oxygen saturation level was 90% on room air. Pulmonary and airway auscultation revealed universally audible vesicular murmurs without adventitious sounds. Laboratory results were as follows: hemoglobin, 18.3 g/dL; hematocrit, 50.4%; total leukocyte count, 6,200/mm^3^ with left shift (53% segmented neutrophils); 15% banded neutrophils; 21% lymphocytes; 1% metamyelocyte; platelet count, 68,000/mm^3^; creatinine, 1.83 mg/dL; urea, 22 m/dL; elevated alanine aminotransferase, 60 IU/L; and aspartate aminotransferase, 120 IU/L. Chest X-ray showed bilateral, diffuse alveolar pulmonary infiltrates.

Binasal cannula oxygen therapy was given, and amoxicillin/clavulanic acid and oseltamivir were prescribed, but the patient’s condition deteriorated progressing to respiratory failure, requiring mechanical ventilation. He was then transferred to an intensive care unit at a local hospital as a suspected case of COVID-19. Signs and symptoms worsened during hospitalization, follow by death 24 hours after the second admission to the health unit. Respiratory secretions had been collected with a nasopharyngeal swab and tested negative for SARS-CoV-2 using reverse transcription–polymerase chain reaction. Dengue virus and influenza were also ruled out.

According to his wife, the patient was a salesman who sold tools and, for this reason, made business trips to other cities. She was unable to say whether during his last trip he came into contact with areas of forest or woodland, but she remembered he mentioned visiting an abandoned warehouse. This scenario raised a suspicion of HCPS, and blood samples from day 5 of the patient’s illness were evaluated by reverse transcription–polymerase chain reaction for orthohantavirus.^10^ The viral genome was detected and the virus was identified as the *Juquitiba orthohantavirus* genotype (GenBank MW883072), an orthohantavirus commonly associated with HCPS in the southern and southeastern regions of Brazil.

## DISCUSSION

HCPS, a zoonotic disease caused by different species of the genus *Orthohantavirus*, is endemic in several Brazilian regions. Since 1993, more than 2,200 cases of HCPS have been reported in 17 federated units, most of them in the southern, southeastern, and midwestern regions of the country.^11^^–^^13^ With a global case fatality rate of ≈40%, the most frequently reported clinical manifestations of HCPS are fever, fatigue, cough, shortness of breath, muscle pain, headache, vomiting, and diarrhea, all of which very similar to other viral infectious diseases, mainly COVID-19 (Table 1). As observed for HCPS and other neglected or rare infectious diseases, the diagnosis can be missed easily if the disease is not suspected, emphasizing the importance of epidemiological data during a clinical investigation that allows another diagnostic hypotheses to be ruled out.

Hantavirus infection in Brazil is predominantly related to agricultural occupations and any activity that promotes dispersion of aerosols or dust, such when individuals are exposed to or clean places harboring rodents, or during grain transportation, military training, deforestation, agricultural harvesting. These and other occupations place people at risk for acquiring HCPS.^11^^,^^14^

Our patient lived in an urban area of a small municipality in the state of Rio Grande do Sul, a southern region in Brazil, where a single HCPS case had been identified. The patient visited abandoned buildings during a business trip to rural areas of central-western Paraná State and midwestern Santa Catarina State, where HCPS cases are often reported.^11^^,^^15^^,^^16^ Considering that the patient had been isolated at home since his return, one can reasonably suspect a case of imported orthohantavirus infection. According to Fonseca et al.,^12^ 70% of deaths resulting from orthohantavirus in Brazil occur in rural areas, especially in the northern and southern regions. It is worth mentioning that, in recent years, human cases are also being reported in the periphery of urban areas, in places with an insufficient sanitary infrastructure, and with rodent infestations resulting from the unordered growth of human dwellings and deforestation practices.^17^

Imported cases of HCPS are unusual worldwide, and HCPS has been reported especially in countries that are in close geographic proximity, or in travelers to, disease endemic areas.^18^ Considering the continental size of Brazil and the heterogeneous distribution of orthohantavirus infection throughout the country, it Is expected that imported cases of HCPS may occur. According to the Brazilian National Notifiable Diseases Information System,^13^ from 2001 to 2017, 96 HCPS cases were reported outside the region where the infection probably occurred, representing 5.3% (96 of 1,811) of the HCPS cases reported during the same period. Thus, it is essential for early diagnosis that medical practitioners evaluate the presence of risk behaviors and traveling history of acute respiratory syndrome cases, screening those patients for orthohantavirus to reduce the morbidity and mortality caused by these diseases.

Orthohantavirus infections are a growing public health problem and there is currently no therapy or vaccine in global use. Thus, in severe HCPS cases, the use of inotropic agents and careful administration of fluids are strongly recommended to prevent the worsening of pulmonary edema. The prognosis in severe cases of HCPS is poor, and the lethality rate is high, with ≈40% to 50% of patients progressing to death. Early diagnosis and an adequate support therapy can increase survival in 70% to 80% of cases.^19^^,^^20^

Differential diagnosis of HCPS from several infectious diseases, such as dengue, influenza, histoplasmosis, malaria, Q fever, pulmonary leptospirosis, and, more recently, COVID-19, is difficult because of overlapping initial clinical symptoms,^20^ as we have demonstrated (Table 1). It is worth mentioning that, especially in HCPS endemic areas, this highly lethal zoonosis needs to be ruled out in patients with clinical manifestations compatible with other respiratory infections such as influenza and COVID-19. Recently, in Europe, a confirmed case of *Puumala orthohantavirus* infection was reported in a health-care worker with a misdiagnosis of COVID-19.^21^

Although radiological changes in the lungs of patients with HCPS can be confused with COVID-19, chest X-ray findings showing bilateral, irregular alveolar infiltrates with air bronchograms may be suggestive of HCPS.^22^ These findings were described in our patient, who presented an influenza-like illness with an epidemiological history compatible with HCPS.^23^ With regard to high-resolution computed tomography (CT), there is limited information in HCPS cases. CT findings in patients with HCPS are rare, and predominantly note bilateral ground-glass opacities associated with smooth inter- and intralobular septal thickening, similar to COVID-19 CT findings.^22^^–^^25^

In our patient, in view of the negative result for COVID-19, a presumptive diagnosis of HCPS was made based on his epidemiological history and clinical manifestations associated with laboratory findings. Some biochemical results can support the diagnosis of a suspected case of HCPS, because the blood counts show, in most cases, hemoconcentration (hematocrit > 49%), thrombocytopenia, and an elevated leukocyte count with left shift—uncharacteristic results for viral infections.^6^^,^^11^^,^^15^

As noted, patients with HCPS can progress rapidly to respiratory failure. Therefore, it is important to investigate patients’ exposure and activities in affected regions, as well as incursions to endemic areas. Furthermore, although orthohantavirus and SARS-CoV-2 co-infection has not yet been reported, it is necessary to consider the possibility of co-infection, as already observed with other respiratory viruses such as influenza, rhinovirus/enterovirus, parainfluenza, metapneumovirus, and influenza B virus.^26^^–^^28^

In the face of the COVID-19 pandemic, our patient highlights the importance of differential diagnoses, especially in those individuals who have a travel history to endemic areas during the previous 60 days and who present with manifestations compatible with HCPS, such as respiratory failure and dry cough.
